# Targeting the KLF5/PI3K/AKT axis as a therapeutic strategy to overcome neoadjuvant chemoresistance in colorectal cancer

**DOI:** 10.3389/fimmu.2025.1593639

**Published:** 2025-07-15

**Authors:** Meiling Gao, Jiahao Qian, Ping Xia, Wancheng Liu, Yunjia Jiang, Yuchang Xia, Xin Yao, Qingqing Jiao, Minggang Wei

**Affiliations:** ^1^ Central Research Laboratory, The First Affiliated Hospital of Soochow University, Suzhou, China; ^2^ Department of Traditional Chinese Medicine, The First Affiliated Hospital of Soochow University, Suzhou, China; ^3^ Department of Clinical Laboratory, Qilu Hospital of Shandong University, Jinan, Shandong, China; ^4^ Department of Dermatology, The First Affiliated Hospital of Soochow University, Suzhou, China

**Keywords:** colorectal cancer, oxaliplatin resistance, KLF5, GDC-0941, neoadjuvant chemotherapy, PI3K/Akt pathway, chemoresistance

## Abstract

**Background:**

Oxaliplatin-based neoadjuvant chemotherapy (NAC) is the standard treatment for advanced colorectal cancer (CRC), yet resistance to NAC poses a significant clinical challenge.

**Methods:**

To investigate the mechanisms of chemoresistance, we analyzed single-cell RNA sequencing (scRNA-seq) data from CRC patients undergoing NAC. Comprehensive analyses, including InferCNV, differentially expressed genes analysis, pathway enrichment, cell communication, and SCENIC were performed. High-throughput drug screening identified potential therapeutic candidates targeting chemoresistance pathways, and the efficacy of targeting the KLF5/PI3K/AKT axis in combination with oxaliplatin was explored in animal models.

**Results:**

NAC effectively reduced tumor burden and enhanced T_NK cell infiltration in responsive tumors. Notably, NAC-resistant cell clusters exhibited activation of fatty acid-related metabolic pathways and demonstrated limited immune infiltration. Transcriptional analysis identified KLF5 as a potential driver of chemotherapy resistance. Based on these findings, we developed a KLF5 regulon-associated risk score model with significant potential for predicting CRC patient prognosis. Mechanistically, KLF5 activation of the PI3K/AKT pathway conferred chemoresistance in CRC cells. Through high-throughput screening, GDC-0941, a PI3K/AKT inhibitor, emerged as a promising therapeutic agent that synergistically enhanced oxaliplatin efficacy and overcame resistance in preclinical models.

**Conclusions:**

Targeting the KLF5/PI3K/AKT axis may enhance chemotherapy efficacy and overcome drug resistance in CRC.

## Highlights

What is already known on this subject:

- Neoadjuvant chemotherapy (NAC), in combination with surgery and/or immunotherapy, is the standard treatment for metastatic colorectal cancer (CRC). However, nearly half of the patients develop chemoresistance to these therapies.
- The immunosuppressive tumor microenvironment (TME) plays a critical role in the development of chemoresistance. Additionally, malignant epithelial cells can undergo remodeling during NAC, but the dynamics of this process and its impact on chemoresistance remain poorly understood.
- The KLF5 transcription factor and the PI3K/AKT signaling pathway are known to promote tumor progression. However, the exact mechanisms through which they contribute to chemoresistance are still not fully understood.

What this study adds:

- Immune and epithelial cell alterations in p-MMR/MSS CRC undergoing NAC.
- Metabolic activity and PI3K/AKT pathway alterations contribute to NAC resistance.
- KLF5 regulon drives chemoresistance and predicts CRC patient prognosis.
- GDC-0941 combined with oxaliplatin overcomes chemoresistance by enhancing immune response.

How this study might affect research, practice, or policy:

These findings offer valuable insights for analyzing NAC-resistant CRC and provide a foundation for developing combination therapies to improve patient responses to NAC. By targeting the KLF5/PI3K/AKT axis, we may enhance the effectiveness of existing treatments through the activation of the immune response.

## Introduction

Colorectal cancer (CRC) is among the most prevalent and lethal malignancies of the digestive system (1). Notably, the majority of CRC patients manifest the microsatellite-stable (MSS) subtype, and immune checkpoint inhibitors are generally ineffective in directly treating these patients. Therefore, neoadjuvant chemotherapy (NAC) in combination with surgery and/or immunotherapy, has become the standard treatment for metastatic CRC (2–4). However, nearly half of the patients eventually develop resistance to treatment, posing a significant challenge to optimizing therapeutic strategies for metastatic CRC patients.

The tumor microenvironment (TME) is a complex ecosystem that comprises various cell types, such as immune cells, fibroblasts, stromal cells, and epithelial cells. Each of these integrated effects on tumor progression and response to therapy (5). Recent advances in the single-cell RNA sequencing (scRNA-seq) analysis of CRC patients undergoing NAC have revealed that the immune phenotype frequently shifts toward immunosuppression in patients who develop resistance to NAC (6–8). However, the role of epithelial cells in driving therapy resistance has been largely overlooked. We hypothesize that following NAC treatment, epithelial cells undergo remodeling that not only confers resistance to chemotherapy but also facilitates immune evasion. To address this critical knowledge gap, we developed a novel pipeline to comprehensively investigate epithelial cell dynamics in NAC-treated CRC patients and to identify potential therapeutic strategies for overcoming resistance.

In our study, we identified KLF5 as a key transcription factor that drives chemoresistance in CRC. Mechanistically, KLF5 activates the PI3K/AKT signaling pathway which subsequently promotes immune evasion. Additionally, GDC-0941, a selective PI3K/AKT inhibitor, significantly enhanced the efficacy of oxaliplatin by reactivating the immune system and promoting immune-mediated tumor cell killing. Furthermore, we developed a gene signature model based on the KLF5 regulon that demonstrated independent prognostic value for predicting clinical outcomes in CRC patients, potentially offering a valuable tool for treatment stratification.

## Materials and methods

### Data acquisition and preprocessing

scRNA-seq data from CRC patients were obtained from the Gene Expression Omnibus (GEO) database under accession numbers GSE178318, GSE225857, and GSE178341. Samples of normal tissue, non-NAC (surgery-alone treated) tumors, and tumors treated with NAC were selected from these datasets for further analysis. All patients were confirmed to have MSS tumors (**Supplementary Table S1**).

Unique molecular identifier (UMI) matrices for each sample were integrated, and batch effects were removed by the Harmony R package (version 1.2.0) (9). Subsequent analyses were performed using Seurat (version 5.1.0) (10) with default parameters unless otherwise specified in the methods section. Data normalization was performed using the ‘NormalizeData’ function with natural-log-transformed normalization. Based on the normalized matrix, highly variable genes (top 2000) were identified using the ‘FindVariableFeatures’ function with the ‘vst’ method. Principal components analysis (PCA) was performed on the integrated matrix for dimensionality reduction, and cell clusters were identified using the ‘FindClusters’ function based on the top 40 principal components (PCs) with a resolution of 0.8. The resulting clusters were annotated based on the expression of already known marker genes (11). Clusters were visualized using Uniform Manifold Approximation and Projection (UMAP) for dimensionality reduction and spatial representation.

### Identification of neoadjuvant-chemotherapy-resistant clusters

To further characterize the NAC-resistant cluster, InferCNV (version 1.20.0, https://github.com/broadinstitute/inferCNV) was applied to infer copy number variations (CNVs). A reference set consisting of 1,000 randomly selected normal epithelial cells with near-diploid copy number profiles was used. Additional cells exhibiting significant CNVs were identified and considered as malignant epithelial cells. These cells were subsequently subjected to dimensional regression and re-clustering, providing a detailed characterization of malignant epithelial cell clusters.

### Differential analysis and functional enrichment

To identify marker genes for each cluster and differentially expressed genes (DEGs) between clusters, the ‘FindMarkers’ function in Seurat V5 was used. The Gene Ontology (GO) terms and KEGG pathways enriched by the DEGs were identified using the ‘clusterProfiler’ R package (version 4.10.1) (12–15).

The ‘ScMetabolism’ R package (version 0.2.1) (6) was utilized to calculate metabolism-related pathway scores for each cluster.

The LINCS L1000 CMAP signatures represent mRNA expression profiles of cell lines following small molecule perturbations. We collected 30,970 downregulated gene sets that were differentially expressed in response to small molecule perturbations from Harmonizome 3.0 (https://maayanlab.cloud/Harmonizome/). Then we performed Gene Set Enrichment Analysis (GSEA) to determine whether the marker genes were spatially enriched in these signatures. According to the results of GSEA, the top 5 pathways with the highest normalized enrichment scores (NES) were expected to inhibit the marker genes.

### Construction of cell communication and regulatory networks

CellChat (version 1.6.1) (16), a widely used computational tool for cell communication analysis, was performed to infer cell cluster communication networks. We compared the TME communication networks between NAC-resistant and NAC-sensitive epithelial clusters. Based on ligand-receptor interactions, intercellular communication networks among clusters were inferred to identify key differences between NAC-resistant and NAC-sensitive clusters.

SCENIC (version 1.3.1) (17) was applied to construct the regulatory network and identify constitutively activated regulons that drive NAC resistance. The activity of each regulon was quantified using the AUCell algorithm, which calculates the area under the curve (AUC) scores for single-cell data.

### Prognostic model construction and inhibitor screening

The mRNA expression profiles of COAD (colon adenocarcinoma) and READ (rectal adenocarcinoma) were obtained from the TCGA database (https://www.cancer.gov/ccg/research/genome-sequencing/tcga). A total of 508 patients with complete clinical information were included, comprising 406 alive and 102 deceased cases. Patients with missing survival data were excluded from survival analysis. No missing values were present in the gene expression dataset. Detailed patient characteristics, including demographic and pathological features, are provided in **Supplementary Table S3**. Univariate Cox regression analysis was performed to identify genes associated with prognosis within the KLF5 regulon. LASSO regression and multivariate Cox regression were subsequently applied to refine the gene set and construct a risk score model. CRC patients were stratified into high-risk and low-risk groups based on their risk scores. Kaplan-Meier (KM) survival analysis was conducted to evaluate differences in overall survival between the two groups. Finally, the GSE39582 and GSE17536 datasets (18, 19) were used to validate the robustness and predictive accuracy of the risk model.

The ESTIMATE R package (version 1.0.13, https://github.com/oicr-gsi/ESTIMATE) was used to calculate tumor purity and immune scores, which were compared between high-risk and low-risk groups based on the risk score model.

The oncoPredict R package (version 1.2) (20) was employed to predict the IC50 values of inhibitors for each patient based on drug sensitivity data from the GDSC2 database (21) (https://www.cancerrxgene.org/). Inhibitors exhibiting significant negative correlations with risk scores were prioritized for further investigation.

### Cell lines and reagents

MC38 murine colon adenocarcinoma and HCT116 cell lines were purchased from MEIXUAN Biological Science & Technology (Shanghai, China), and cultured in 1640 medium supplemented with 10% fetal bovine serum (FBS), 100 U/mL penicillin, and 50 μg/mL streptomycin. Oxaliplatin (Cat# HY-17371), and GDC-0941 (Cat# HY-50094) were purchased from MedChemExpress. Annexin V-FITC/PI Apoptosis Detection Kit was purchased from Vazyme (Cat# A211-01).

### Mouse experiments

Female C57BL/6N and nude mice (6–8 weeks old) were purchased from Beijing Vital River Laboratory Animal Technology Co. Ltd. All mice were housed in the Laboratory Animal Center of Suzhou Medical College, Soochow University, under specific pathogen-free (SPF) conditions. Age- and sex-matched mice were randomly assigned to groups, with at least 5 mice per group.

A total of 5 × 10^5^ MC38 cells suspended in 100 μL PBS were injected into the tail vein of C57BL/6N or nude mice to establish a lung metastasis model. Seven days after tumor injection, mice were treated with oxaliplatin (5 mg/kg, intraperitoneally once per week), ML264 (25 mg/kg, intraperitoneally twice a week), GDC-0941 (25 mg/kg, daily via oral gavage), or vehicle control. Lung metastases were assessed and evaluated through Hematoxylin and eosin (H&E) staining, while survival rates were analyzed using KM survival curves. H&E staining was performed by FuncBio (Suzhou) Biotechnology Co.Ltd.

### Western blot

A total of 1 × 10^6^ cells were seeded in 6-well plates and treated with 10 μg/mL Oxaliplatin and 6.25 μg/mL GDC-0941 for 48 hours. After incubation, the cells were harvested and lysed using RIPA lysis buffer (Beyotime, P0013C) supplemented with 1 mM PMSF (Beyotime, ST506) to prevent protein degradation. The cell extracts were subjected to immunoblotting with the indicated antibodies to evaluate the expression levels of target proteins. The following antibodies were used: Anti-AKT1/2/3 (Abmart, T55561S), Anti-Phospho-Akt (Ser473) (Abmart, T40067), Anti-β-Actin (Abmart, P30002).

### CCK8 assay

A total of 8,000 cells were seeded in 96-well plates and treated with oxaliplatin at nine concentrations prepared in a 2-fold serial dilution series, starting from an initial concentration of 100 μg/mL. After 48 hours of incubation, cell viability was assessed using the CCK-8 Cell Counting Kit (Vazyme, A311-01) following the manufacturer’s instructions. The absorbance was measured at OD450 nm using an ELISA plate reader.

### Establishment of oxaliplatin-resistant HCT116 cell line

The HCT116 cell line was cultured in RPMI-1640 medium containing low concentrations of oxaliplatin, with the drug concentration gradually increased step by step. The cells were continuously exposed to oxaliplatin until they could proliferate normally in RPMI-1640 medium containing 25 μg/mL oxaliplatin.

### Statistics

The scRNA-seq data and TCGA mRNA expression matrix analyses were performed using R software (version 4.3.0). Comparisons between the two groups were conducted using the Wilcoxon rank-sum test. The data were shown as mean ± SD, and data visualization was performed using GraphPad Prism (version 8.4.0). For multiple-group comparisons, one-way ANOVA was applied. Statistical significance was defined as follows: *P* < 0.05 = “*”, *P* < 0.01 = “**”, *P* < 0.001 = “***”, *P* < 0.0001 = “****”, *P* > 0.05 = ns, not significant.

## Results

### Systematic scRNA-seq analysis of the TME in CRC patients with NAC

To investigate how changes in the cellular composition of the TME respond to NAC in CRC patients, we collected scRNA-seq data from 82 samples (8, 22, 23), divided into four groups: 36 adjacent normal samples, 5 NAC-treated adjacent normal samples, 32 untreated tumor samples (Non-NAC, -NAC tumor group), and 9 tumor samples from CRC patients undergoing NAC (**Supplementary Table S1**).

**Figure 1:**
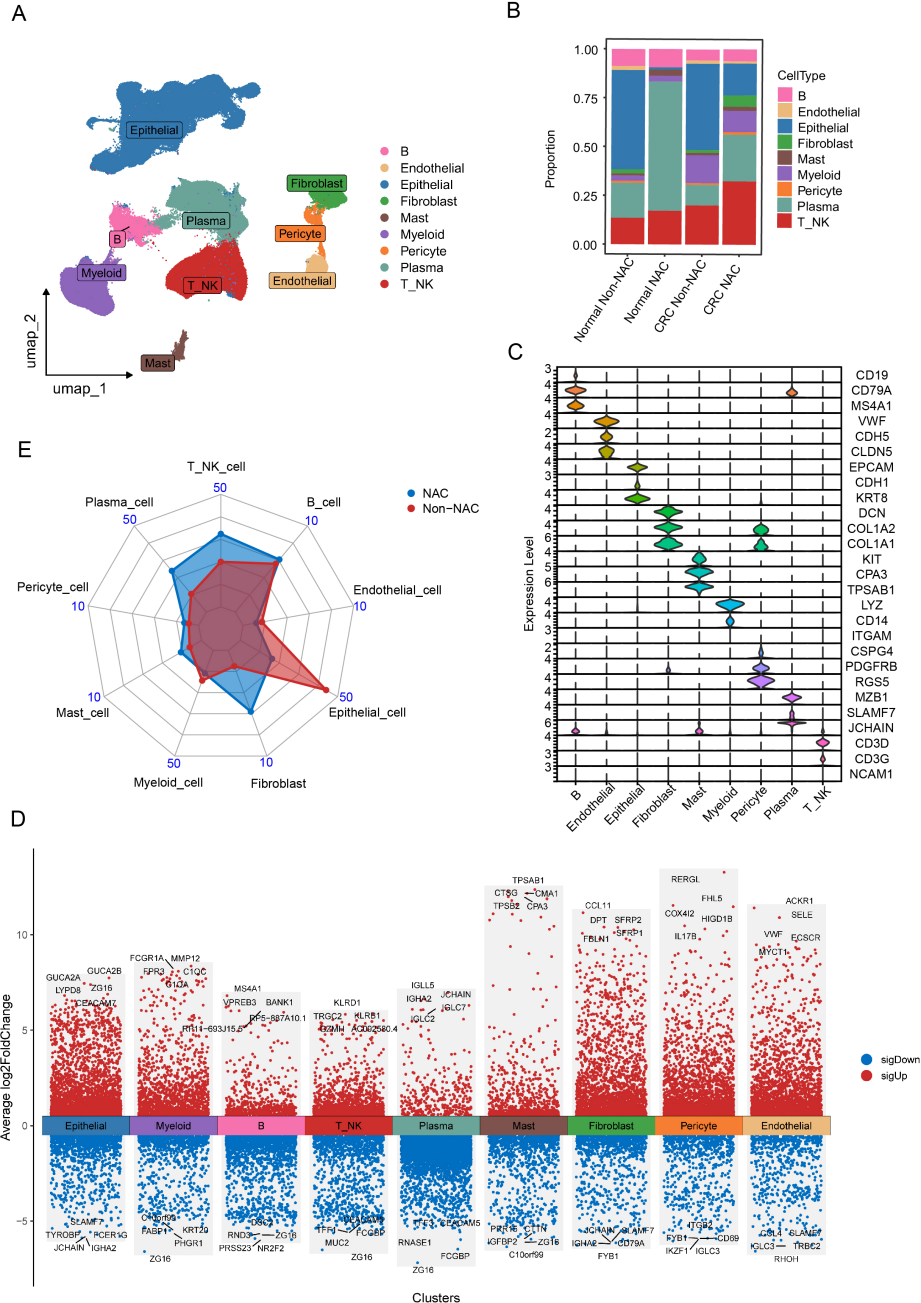
Single-cell atlas of CRC patients undergoing NAC treatment. **(A)** UMAP plot of broad cell clusters from all samples. **(B)** Boxplot showing the relative frequencies of different cell clusters stratified by CRC patient treatment strategies. **(C)** Violin plots illustrating the expression patterns of marker genes across the identified cell clusters. **(D)** Heatmap of DEGs identified among the nine major cell clusters, generated using the “FindMarkers” function in Seurat V5 (parameters: min.pct = 0.15, logfc.threshold = 0.2). **(E)** Radar chart comparing the proportion of cell clusters between NAC and Non-NAC groups.

After quality control and batch effect removal (see Materials and Methods), the transcriptomes of 388780 high-quality single cells were obtained. Next, we performed normalization, and PCA, and visualized the resulting cell clusters using UMAP. To further characterize the cell clusters, we analyzed the marker genes of all clusters and identified nine broad cell types, containing T_NK cells (T cells and NK cells), B cells, myeloid cells, epithelial cells, endothelial cells, mast cells, pericytes, plasma, and fibroblasts (**Figures 1A–D**) While NAC induced no significant changes in adjacent normal tissues, tumors treated with NAC exhibited an increased proportion of immune cells and a decreased proportion of epithelial cells compared to untreated tumors (**Figures 1B, E**). These findings suggest that chemotherapy exerts a potent tumor-killing effect and promotes immune cell infiltration. Additionally, significant inter-sample heterogeneity was observed based on the changes in cellular composition across samples (**Supplementary Figure S1**).

### Identification and characterization of NAC-resistant cell clusters

**Figure 2:**
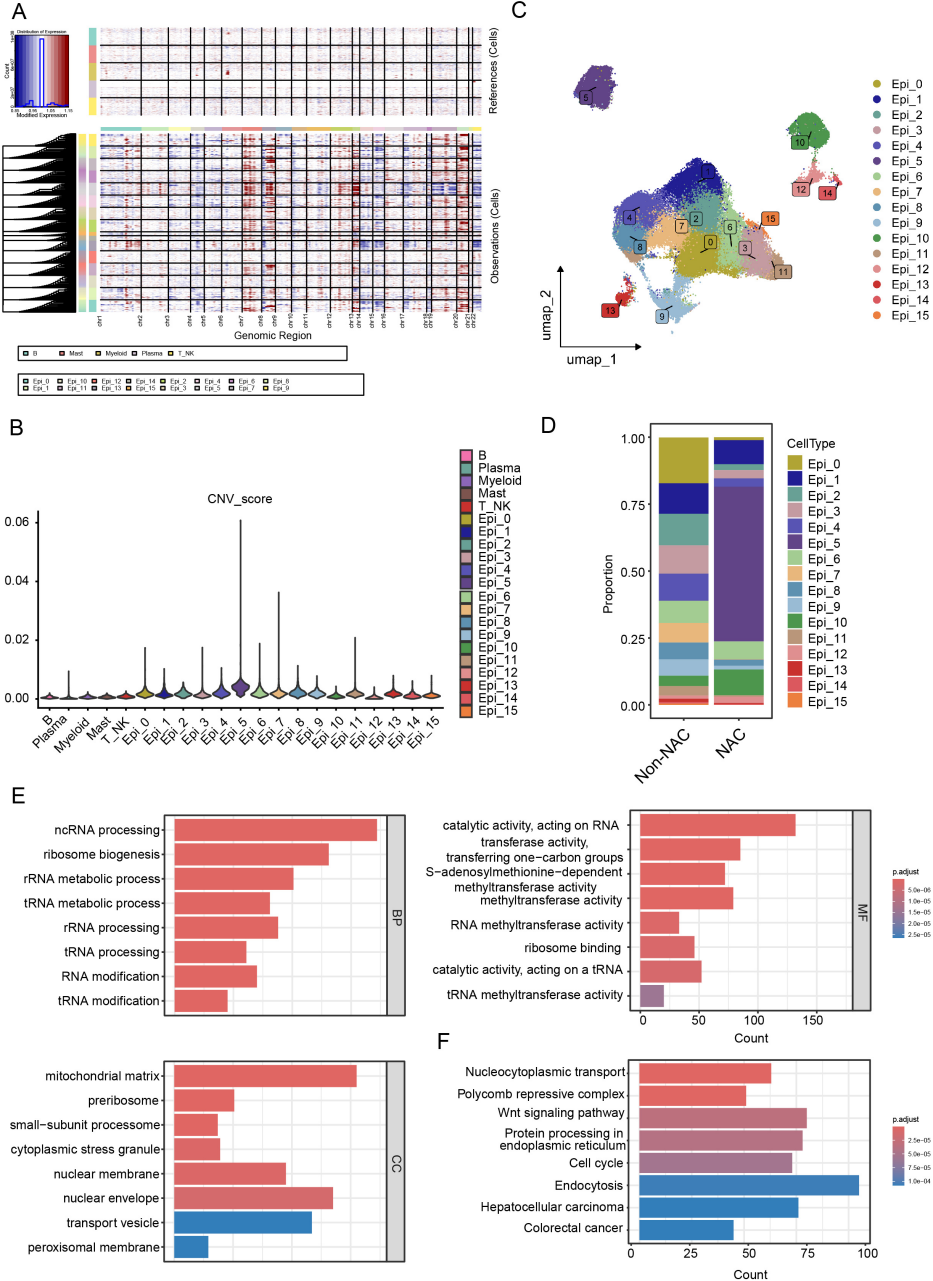
Identification and characterization of NAC-resistant cell clusters. **(A, B)** Identification of malignant epithelial cells using the InferCNV algorithm. **(A)** InferCNV analysis reveals CNV profiles across all chromosomes, and **(B)** cells with high CNV scores were identified as malignant compared to immune cell types. **(C)** UAMP plot of malignant epithelial cell clusters. **(D)** Boxplot illustrating the proportions of tumor cell clusters in NAC and non-NAC groups. **(E)** GO term enrichment analysis reveals key biological processes in Epi_5. **(F)** KEGG pathway analysis identifies activated signaling pathways in Epi_5.

We used InferCNV to analyze epithelial cells, classifying them into malignant and normal cells based on CNV profiles (**Figures 2A, B**). After dimensionality reduction and re-clustering, we identified 16 stable tumor cell clusters (**Figure 2C**), which displayed diverse distributions following NAC treatment (**Figure 2D**), indicating that these clusters may be associated with therapy resistance. Among them, the Epi_5 cluster showed the highest proportion of surviving cells in patients after NAC (**Figure 2D**).

GO and KEGG enrichment analyses of the marker genes from the Epi_5 cluster (**Supplementary Table S2**) revealed predominant enrichment in RNA modification and metabolism-related pathways (**Figures 2E, F**, **Supplementary Figure S2A**). Given the strong correlation between tumor metabolism and therapy resistance (24), we conducted ‘ScMetabolism’ analyses across all tumor lineages. Our results revealed the significant upregulation of fatty acid metabolism in the Epi_5 cluster (**Supplementary Figure S2B**). To further investigate whether the Epi_5 cluster represents a therapy-resistant stem-like population, we analyzed the expression profiles of CRC stem cell gene markers (CSCs) before and after NAC (25). The results confirmed that the Epi_5 cluster exhibited markedly higher stemness scores compared to other epithelial clusters (**Supplementary Figures S2C–E**). Additionally, to identify potential drugs able to suppress Epi_5 gene expression and restore sensitivity to NAC, we performed CAMP L1000 signature analysis. The top 5 drugs were displayed in **Supplementary Figure S2F**, among which ATPase inhibitors and PI3K inhibitors demonstrated the potential to overcome NAC resistance in the Epi_5 cluster (**Supplementary Figure S2F**). Considering the characteristics of the Epi_5 cluster, multiple mechanisms are likely involved in contributing to NAC resistance. Targeting energy metabolism and the PI3K/AKT pathway may provide effective strategies to overcome drug resistance.

### Cellular communication in the TME

**Figure 3:**
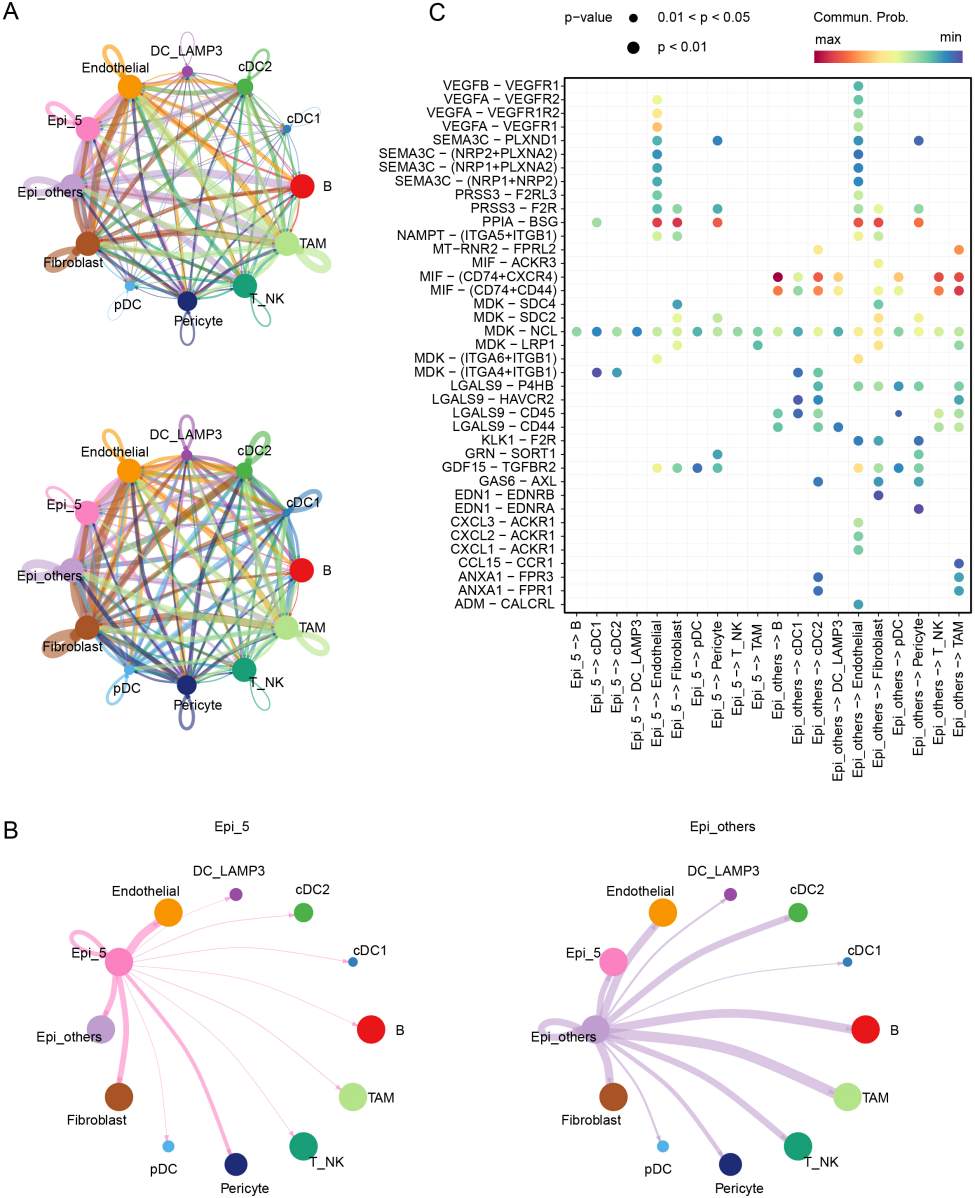
Cellular communication in the TME. **(A)** Communication counts and weight among various cell clusters in the TME. **(B)** Communication weights between Epi_5 and Epi_others and their interactions with other cell clusters in the TME. **(C)** Signaling pathways mediating communication within and between Epi_5 and Epi_others.

To investigate the interactions between epithelial cells within the tumor microenvironment, we employed CellChat for analysis. Compared to the NAC-resistant epithelial cells, other epithelial cells exhibited enhanced communication with immune cells, such as tumor-associated macrophages, dendritic cells, B cells, and T_NK cells in the tumor immune microenvironment (**Figures 3A, B**). Receptor-ligand analysis revealed highly active secretory protein pathways, such as VEGFA-VEGFR1/VEGFR2 and SEMA3C-PLXND1, in the NAC-resistant epithelial cluster (**Figure 3C**, **Supplementary Figures S3A, B**).

During treatment, in addition to directly killing tumor cells, chemotherapy can induce tumor cell stress, triggering the release of cell death-associated molecular patterns (CDAMPs), which are recognized by immune cells, activating an anti-tumor immune response to eliminate residual tumor cells and prevent metastasis and recurrence (26). However, in CRC patients, particularly those with pMMR/MSS status, tumor immune infiltration is significantly limited (27, 28). To further clarify the immunological mechanism in patients corresponding to the Epi_5 epithelial cluster, we divided patients following NAC into two groups based on Epi_5 composition (**Supplementary Figure S3C**). Our analysis revealed that patients with low Epi_5 cluster representation demonstrated significantly higher T and NK cell infiltration and significant enrichment of antigen processing and presentation pathways (**Supplementary Figures S3D, E**). These findings suggest that enhanced angiogenesis and impaired immune cell infiltration are key factors contributing to NAC resistance.

### KLF5 regulon specifically activated in the Epi_5 cluster

**Figure 4:**
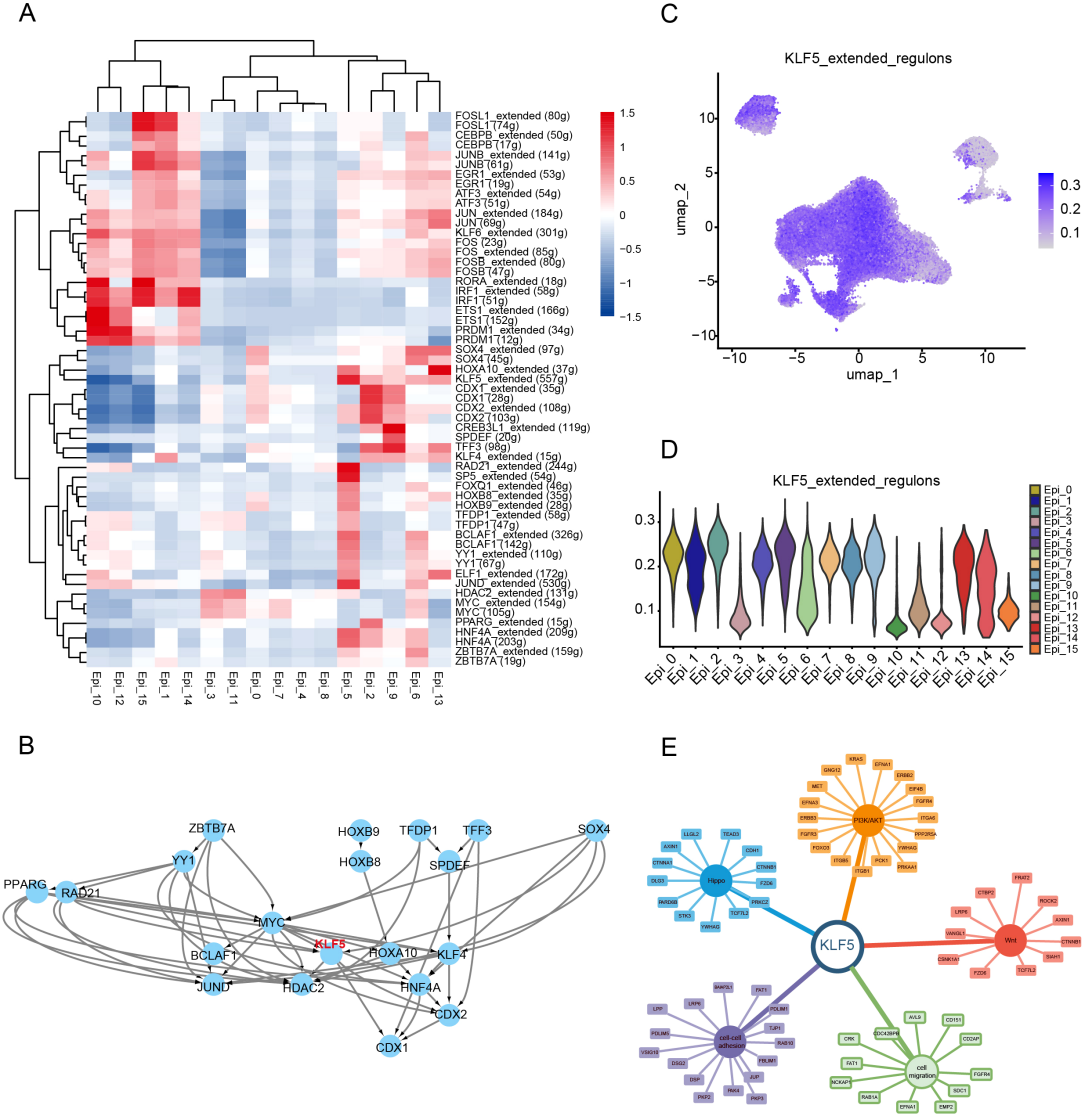
KLF5 regulon specifically activated in the Epi_5 cluster. **(A)** Identification of spatially activated transcription factors in Epi_5 using the SCENIC algorithm. **(B)** Interaction and regulatory network of transcription factors identified in Epi_5. **(C, D)** UMAP visualization **(C)** and Violin plot showing the AUCell scores of KLF5 regulon activity in various cell clusters. **(E)** Regulatory network between KLF5 and its target genes, including cell migration, Wnt signaling, Hippo signaling, and PI3K/AKT pathways.

Considering the role of transcription factors in gene expression and regulation, we used SCENIC algorithms to calculate the specifically activated transcription factors in the Epi_5 cluster (**Figure 4A**). The RAD21, SOX4, and KLF5 regulons were significantly upregulated in the Epi_5 cluster (**Figure 4A**), with KLF5 positioned at the center of the network (**Figure 4B**). To identify the key transcription factor driving chemotherapy resistance, we integrated the SCENIC results with a comprehensive literature review. KLF5 regulon was recognized as a center molecule for further analysis (**Figures 4C, D**). Subsequently, we constructed an interaction network for downstream targets of KLF5. Based on this analysis, we successfully established a KLF5-centered regulatory network associated with chemotherapy resistance mechanisms, including pathways related to cell-cell adhesion (29), cell migration (30), Wnt signaling (31, 32), Hippo signaling (33), and PI3K/AKT pathways (34, 35) (**Figure 4E**).

### Exploring therapeutic strategies for NAC-resistant patients driven by the KLF5 regulon

**Figure 5:**
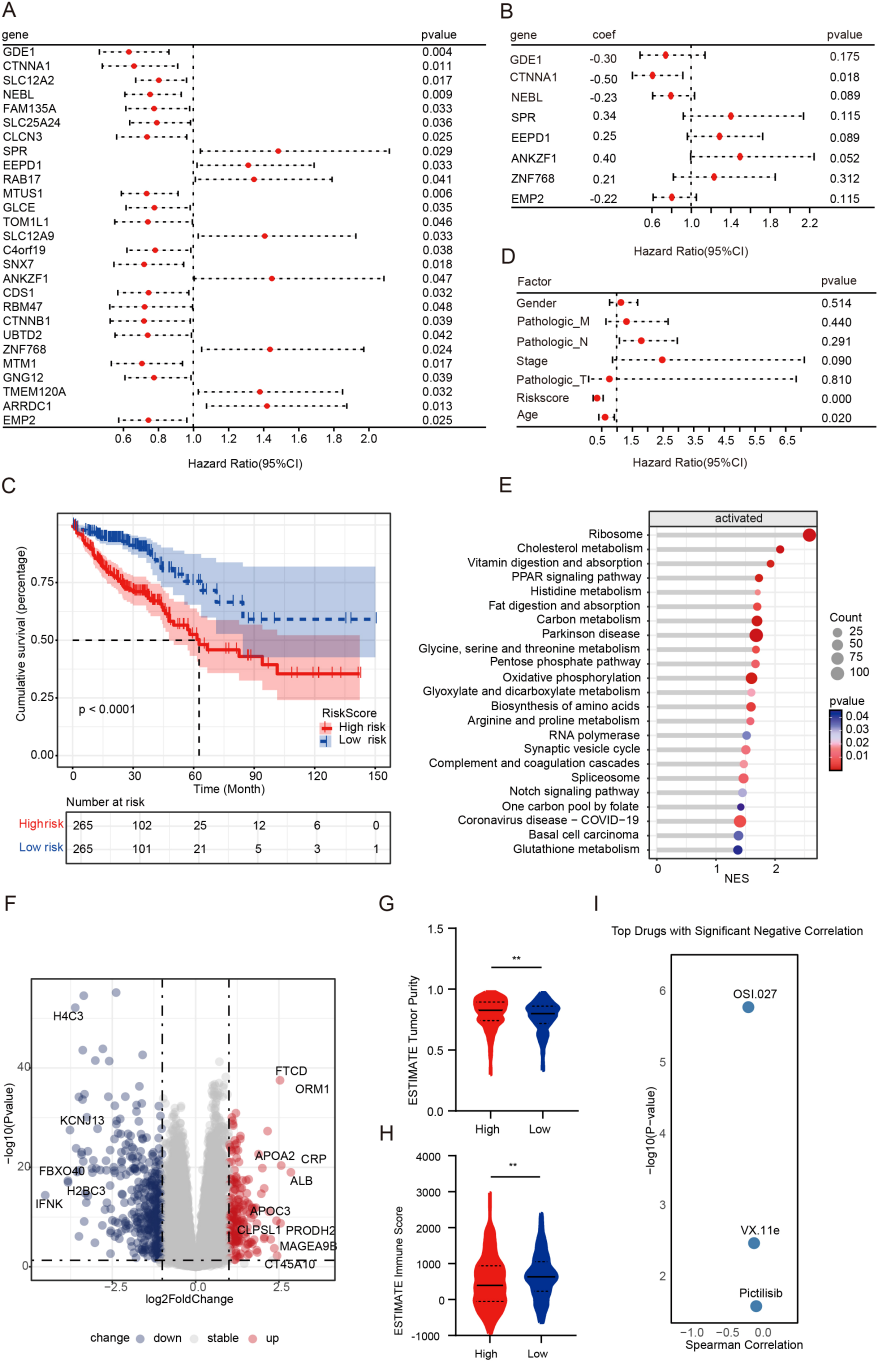
Exploring therapeutic strategies for NAC-resistant patients driven by the KLF5 regulon. **(A, B)** Univariate and multivariate Cox regression analyses of the KLF5 regulon, respectively. **(C)** Kaplan-Meier survival analysis of risk score groups, with patients divided into high- and low-risk groups based on the median risk score. **(D)** Univariate Cox regression analyses of clinical factors. **(E)** GSEA analysis of upregulated genes in the high-risk score group. **(F)** Volcano plot illustrating DEGs between high- and low-risk score groups. **(G, H)** Tumor purity and immune scores were calculated using the ESTIMATE algorithm. Data were shown as mean ± SD. The statistical significance was determined by the Wilcoxon test (**p < 0.01). **(I)** Riskscore-related drug sensitivity screening.

Given the strong relationship between therapeutic outcomes and prognosis, we constructed a prognostic model based on the KLF5 regulon using univariate and multivariate COX regression analyses (**Figures 5A, B**). Survival analysis demonstrated that the risk score model was highly significantly associated with patient prognosis (*P* < 0.0001, **Figure 5C**). To evaluate its robustness, the GSE39582 and GSE17536 datasets were used to validate this model, where it consistently demonstrated strong prognostic correlations (*P <*0.05), confirming its reliability across different datasets (**Supplementary Figure S4C**). Additionally, univariate Cox regression incorporating clinical factors such as tumor stage, gender, age, and risk score showed that the risk score model was an independent prognostic factor for CRC patients, outperforming other clinical variables (**Figure 5D**). Moreover, a nomogram was constructed based on the risk score and clinical factors, providing an effective tool for individualized survival prediction (**Supplementary Figures S4D, E**). The predictive performance of the model was further validated using ROC curve analysis, which yielded an AUC > 0.7, indicating high predictive accuracy (**Supplementary Figure S4F**). These results highlight the robustness and clinical utility of our KLF5 regulon-based risk model in independently predicting CRC patient prognosis.

To further characterize the two risk score groups and explore their differences, we performed differential gene expression and pathway analysis between the high- and low-risk score groups. Our findings revealed that the high-risk group was characterized by significant upregulation of pathways, including metabolism-related pathways, Notch signaling pathways, and PPAR signaling pathways (**Figures 5E, F**). Immediately thereafter, we calculated tumor purity and immune scores using the ESTIMATE algorithm. We found that the high-risk score group displayed significantly higher tumor purity and lower immune infiltration (**Figures 5G, H**). To advance clinical translational research, we investigated potential inhibitors targeting poor-prognosis CRC patients. According to the screening results, OSI-027, VX-11e, and pictilisib (GDC-0941) showed significant negative correlations with risk scores (*P* < 0.05, **Figure 5I**). OSI-027 and GDC-0941 are PI3K/AKT/mTOR pathway inhibitors, suggesting that inhibition of the activated PI3K/AKT/mTOR pathway may overcome drug resistance.

### GDC-0941 enhances chemosensitivity by inhibiting the PI3K/AKT signaling pathway

**Figure 6:**
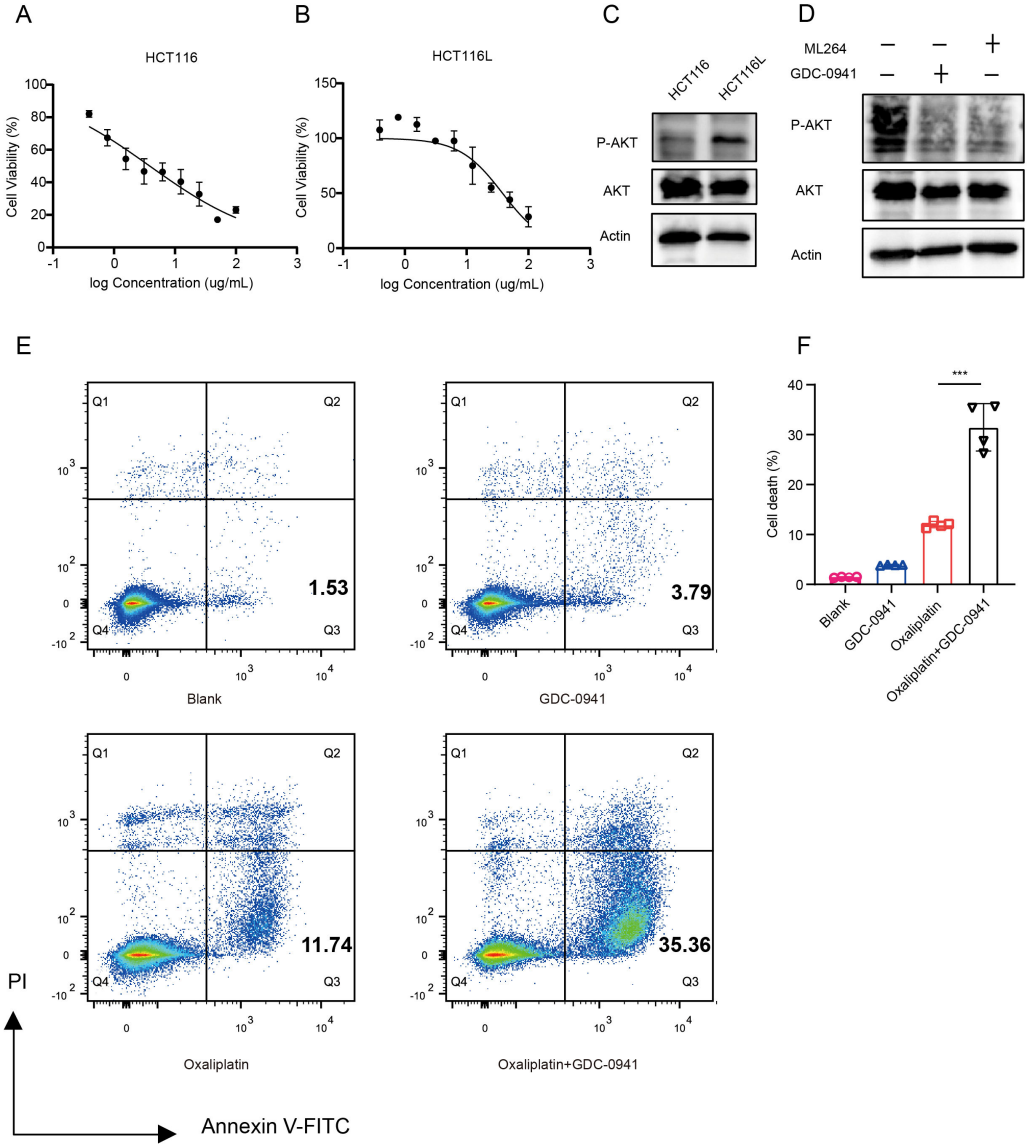
GDC-0941 enhances chemosensitivity by inhibiting the PI3K/AKT/mTOR signaling pathway. **(A, B)** IC50 analyses of HCT116 **(A)** and HCT116L **(B)** cells treated with Oxaliplatin, determined by CCK-8 assay. **(C)** Western blot analysis of total AKT and P-AKT expression levels in HCT116 and HCT116L cells. **(D)** Western blot analysis of AKT and P-AKT expression levels in HCT116 cells treated with GDC-0941 and ML264. **(E, F)** Flow cytometry analysis of apoptosis in HCT116 cells treated with different conditions. Data were shown as mean ± SD. The statistical significance was determined by the Wilcoxon test (***p < 0.001).

Oxaliplatin is one of the most used chemotherapeutic agents for treating advanced CRC patients. To investigate the mechanisms of oxaliplatin resistance, we developed an oxaliplatin-resistant HCT116 (HCT116L) cell line (**Figures 6A, B**, **Supplementary Figures S5A, B**). We observed a significant increase in p-AKT levels in the HCT116L (**Figure 6C**), indicating activation of the PI3K/AKT signaling pathway in drug-resistant cells. To explore the relationship between *KLF5* and p-AKT, we treated HCT116L cells with GDC-0941 or ML264 (KLF5 inhibitor). Western blot analysis revealed that both ML264 and GDC-0941 treatment significantly decreased p-AKT levels (**Figure 6D**). This evidence suggests that KLF5 may act upstream of the PI3K/AKT signaling pathway and potentially contribute to its upregulation, which might promote oxaliplatin resistance.

To evaluate whether GDC-0941 could enhance the efficacy of oxaliplatin, we treated HCT116 cells with oxaliplatin in combination with GDC-0941. We found that the combination of GDC-0941 and oxaliplatin significantly increased the proportion of apoptotic cells compared to oxaliplatin treatment alone (**Figures 6E, F**), demonstrating that GDC-0941 effectively enhances the cytotoxicity of oxaliplatin.

### The combination of GDC-0941 and oxaliplatin enhances antitumor immune responses and suppresses lung metastasis

**Figure 7:**
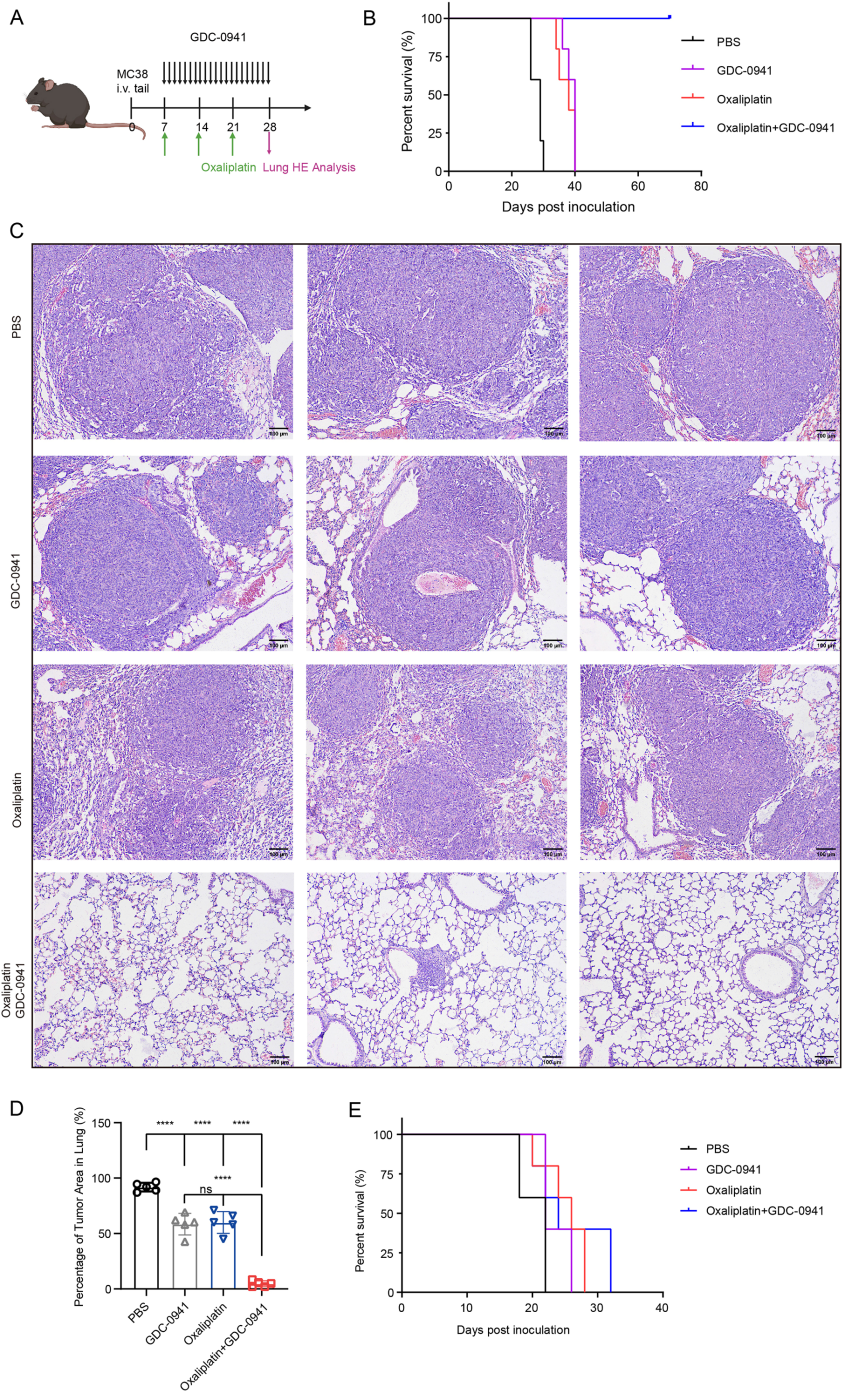
The combination of GDC-0941 and oxaliplatin enhances antitumor immune responses and suppresses lung metastasis. **(A)** Schematic diagram of the experimental design for animal studies. **(B)** KM survival analysis of the four treatment groups in C57BL/6N mice. **(C)** H&E staining of lung tissue from the four groups. **(D)** Quantification of tumor area in the lungs from H&E staining results **(C)**. Data were shown as mean ± SD. The statistical significance was determined by one-way ANOVA (***p < 0.001, ns, not significant: *p*>0.05). **(E)** KM survival analysis of the four treatment groups in nude mice.

To investigate the efficacy of GDC-0941 combined with oxaliplatin *in vivo*, we developed a mouse lung metastasis model by tail vein injection of MC38 cells. Mice were randomly divided into four treatment groups: control, oxaliplatin monotherapy, GDC-0941 monotherapy, and combination therapy (**Figure 7A**). Mice treated with the combination of GDC-0941 and oxaliplatin exhibited a significantly lower mortality rate compared to those receiving monotherapy or no treatment. KM survival analysis revealed that all mice in the combination group survived during the observation period, with a significant difference compared to the control and monotherapy groups (**Figure 7B**). H&E staining indicated that GDC-0941 significantly enhanced the cytotoxicity of oxaliplatin, resulting in a marked reduction in both the number and size of lung tumor nodules (**Figures 7C, D**). Given KLF5’s key regulatory function in NAC resistance, we further investigated whether KLF5 inhibition could similarly overcome oxaliplatin resistance. Indeed, combination therapy with the KLF5 inhibitor ML264 and oxaliplatin demonstrated comparable efficacy in prolonging survival in our lung metastasis model (**Supplementary Figure S6**), further supporting the therapeutic potential of targeting these pathways to enhance oxaliplatin sensitivity.

Given the powerful tumor-eliminating capacity observed, we hypothesized that the immune system might be activated, contributing to increased tumor immune infiltration. To test this hypothesis, we generated a lung metastasis model in nude mice, which lack a functional immune system. KM survival analysis revealed that all nude mice eventually died despite receiving combination therapy (**Figure 7E**). In contrast, immune-competent C57BL/6N mice demonstrated significantly prolonged survival, highlighting the critical role of the immune system in mediating the therapeutic efficacy of the combination treatment. Collectively, these findings suggest that GDC-0941 not only enhances the cytotoxic effects of oxaliplatin but also facilitates immune-mediated tumor elimination.

## Discussion

In this study, we identified chemotherapy-resistant cell clusters using scRNA-seq data from NAC-treated CRC patients. Our findings demonstrate that KLF5 drives chemotherapy resistance by activating the PI3K/AKT signaling pathway. Significantly, treatment with GDC-0941 enhanced CRC cell sensitivity to oxaliplatin and effectively eliminated lung metastasis nodules *in vivo*.

Considering that malignant cells surviving after NAC may have acquired resistance, the Epi_5 cluster was identified as an NAC-resistant cell population. We comprehensively analyzed the Epi_5 cluster and found that the marker gene OTULINL was highly expressed. This gene is homologous to OTULIN, which has been reported to interrupt the mitochondrial apoptotic pathway and induce cisplatin resistance in osteosarcoma (36). Enrichment analysis of the NAC-resistant cluster revealed significant upregulation of fatty acid metabolism pathways and RNA modification processes. Previous studies have shown that m6A RNA modification plays a crucial role in promoting chemotherapy resistance across various cancers (37, 38). Similarly, fatty acid metabolism has been shown to support tumor progression and resistance through degradation and biosynthesis pathways (39). Notably, increased expression of stemness markers and stem-like populations are frequently associated with therapy resistance. The Epi_5 cluster exhibited stemness characteristics, suggesting this population is crucial to NAC treatment outcomes. Our analysis not only identified potential resistance markers but also revealed possible resistance mechanisms, including metabolic reprogramming and alterations in RNA modification. The synergistic effects of these mechanisms are likely to promote tumor cell survival and adaptation under chemotherapeutic pressure. Thus, our comprehensive analysis pipeline of the NAC-resistant cluster provides valuable insights into exploring new therapeutic strategies.

The role of KLF5 in chemoresistance has been increasingly recognized in recent years. Previous studies have established that KLF5 regulates Bcl2 expression, thereby promoting chemotherapy resistance and enhancing cell survival (40). Our study indicated the value of the KLF5 regulon in constructing a prognostic model and identifying potential therapeutic drugs. Among these, GDC-0941 emerged as a promising candidate to overcome KLF5-driven chemotherapy resistance. This finding integrates with previous knowledge that PI3K/AKT inhibition represents a potential therapeutic strategy, as GDC-0941 has shown promising outcomes in clinical trials, particularly in breast cancer and non-small cell lung cancer (41–43). Importantly, clinical evidence suggests that GDC-0941 may enhance immune surveillance by regulating CCL5/CXCL10 (44). Our findings extend the potential application of GDC-0941 to KLF5-mediated chemoresistant CRC, representing a novel therapeutic context for this compound.

Oxaliplatin remains the most used third-generation platinum-based chemotherapy drug. In our experimental tests, we used oxaliplatin-resistant cell lines and observed the activation of the PI3K/AKT pathway, consistent with growing evidence linking AKT activation to platinum resistance. Multiple studies have established that inhibition of the PI3K/AKT pathway can suppress tumor metabolism, particularly lipid metabolism, thereby inhibiting tumor progression (45). Consistent with these findings, we observed enhanced metabolic activity, especially lipid metabolism, in the high-risk score group. Further supporting this connection, single-cell analysis of drug-resistant cell clusters also highlighted the critical role of energy metabolism and the PI3K signaling pathway in mediating drug resistance.

From a clinical perspective, our findings have several important implications. The KLF5 regulon-based risk score we developed could serve as a valuable biomarker for patient stratification, identifying individuals who might benefit most from combination therapy with PI3K inhibitors and conventional chemotherapy. Importantly, our results suggest that patients with tumors showing high KLF5 expression and activated PI3K/AKT signaling may be prime candidates for combination treatment with GDC-0941 and oxaliplatin. Furthermore, the observed metabolic alterations in resistant cells suggest that metabolic targeting could be incorporated into future therapeutic regimens for chemoresistant CRC.

In conclusion, our results provide a strong rationale for targeting the PI3K/AKT signaling pathway, specifically with GDC-0941, as a therapeutic strategy to overcome drug resistance in CRC. By targeting the KLF5/PI3K/AKT axis, this approach addresses a fundamental mechanism of chemoresistance and has the potential to significantly improve outcomes for patients with advanced colorectal cancer.

### Limitations of the present study

Notably, our study was not without limitations. Firstly, the lack of validation using self-collected samples may limit the robustness of our findings. Relying solely on public databases can introduce dataset-specific biases, which may affect the generalizability of our conclusions. Furthermore, future studies should investigate how GDC-0941 modulates immune responses in the context of KLF5/PI3K/AKT inhibition. A deeper understanding of the specific immune pathways activated by this combination therapy could facilitate its integration into immuno-oncology strategies.

### Conclusion

In conclusion, these findings indicated the role of the KLF5/PI3K/AKT axis in chemotherapy resistance. The strong anti-tumor effects observed *in vivo* indicate that the combination of GDC-0941 and oxaliplatin may provide a novel therapeutic strategy in CRC chemotherapy.

## Data Availability

The original contributions presented in the study are included in the article/**Supplementary Material**. Further inquiries can be directed to the corresponding authors.

## Supplementary material

Supplementary Figure 1 Cellular composition across all samples. Proportions of broad cell types identified in all samples, visualized by bar plot.

Supplementary Figure 2 Functional analyses of the Epi_5 cluster. **(A)** DEGs analyses between Epi_5 and all other tumor cell clusters, ranked by log2 fold-change. **(B)** Heatmap of fatty acid metabolism-related signaling pathways across all tumor cell clusters. **(C)** Dotplot showing CSCs in tumor samples before and after NAC. Color intensity indicates expression level while dot size represents the percentage of cells expressing each marker. **(D, E)** UMAP visualization **(D)** and Barplot showing the stemness scores of CSCs in various epithelial cell clusters. **(F)** GSEA plot displaying the top 5 drugs predicted to inhibit the Epi_5 cluster.

Supplementary Figure 3 Communication patterns of all cell clusters. **(A)** Incoming communication patterns of target cells, highlighting the number and types of signals received from other cell clusters. **(B)** Outgoing communication patterns of secreting cells, illustrating the number and types of signals sent to other cell clusters. **(C)** Dotplot illustrating the composition of Epi_5 cells categorized into high and low expression groups. **(D)** Proportions of major cell types within the Epi_5 high and low expression groups. **(E)** GO and KEGG enrichment analyses of differentially expressed genes in T_NK cells comparing Epi_5 high versus low expression groups.

Supplementary Figure 4 Stability validation of the prognostic model. **(A, B)** LASSO regression analysis based on univariate Cox regression results to identify significant predictors. **(C)** Validation of the prognostic model using the GSE39582 and GSE17536 datasets. **(D, E)** Nomogram analysis of risk score and other clinical factors. **(E)** Nomogram showing the probability of survival at 365, 1095, and 1825 days. **(F)** ROC analysis of the prognostic model.

Supplementary Figure 5 Apoptosis analysis of HCT116 and HCT116L cell lines. HCT116 and HCT116L cell lines were treated with 40 μg/ml Oxaliplatin for 48 hours. Apoptotic cells were collected and analyzed using flow cytometry. Data were presented as mean ± SD. The statistical significance was determined by the Wilcoxon test (***p* < 0.01).

Supplementary Figure 6 The combination of ML264 and oxaliplatin suppresses lung metastasis and improves survival. **(A)** Schematic representation of the *in vivo* experimental design: C57BL/6N mice received 5×10^5^ MC38 cells via tail vein injection. Treatment groups received vehicle, oxaliplatin (5 mg/kg, once weekly), ML264 (25 mg/kg, twice weekly), or the combination. **(B)** KM survival curves demonstrating the impact of treatment regimens on mouse survival. The combination of ML264 and oxaliplatin significantly improved survival compared to individual treatments and vehicle control.
