# Supplementary figures and images for: Targeting the KLF5/PI3K/AKT axis as a therapeutic strategy to overcome neoadjuvant chemoresistance in colorectal cancer
Source: Front Immunol. 2025 Jul 15;16:1593639. doi: 10.3389/fimmu.2025.1593639 (PMC12303937; doi:10.3389/fimmu.2025.1593639)

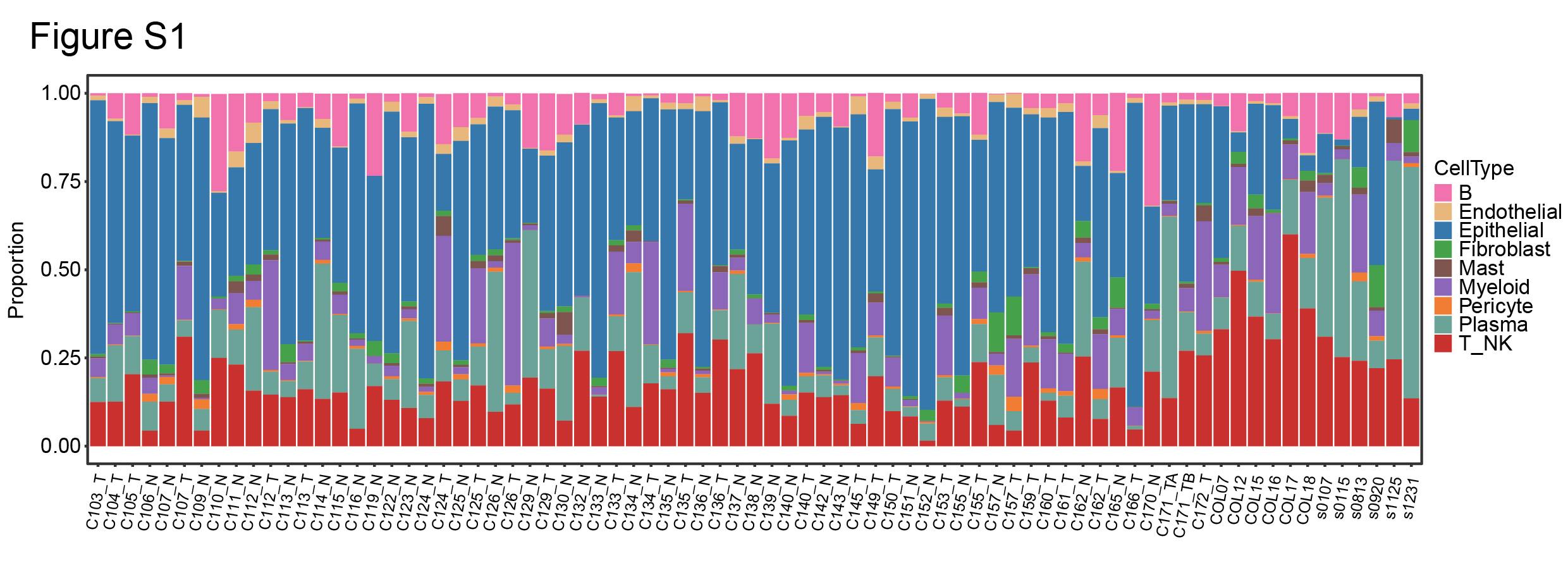

Supplement: Supplementary Figure 1 — Cellular composition across all samples. Proportions of broad cell types identified in all samples, visualized by bar plot. [file Image1.jpg]

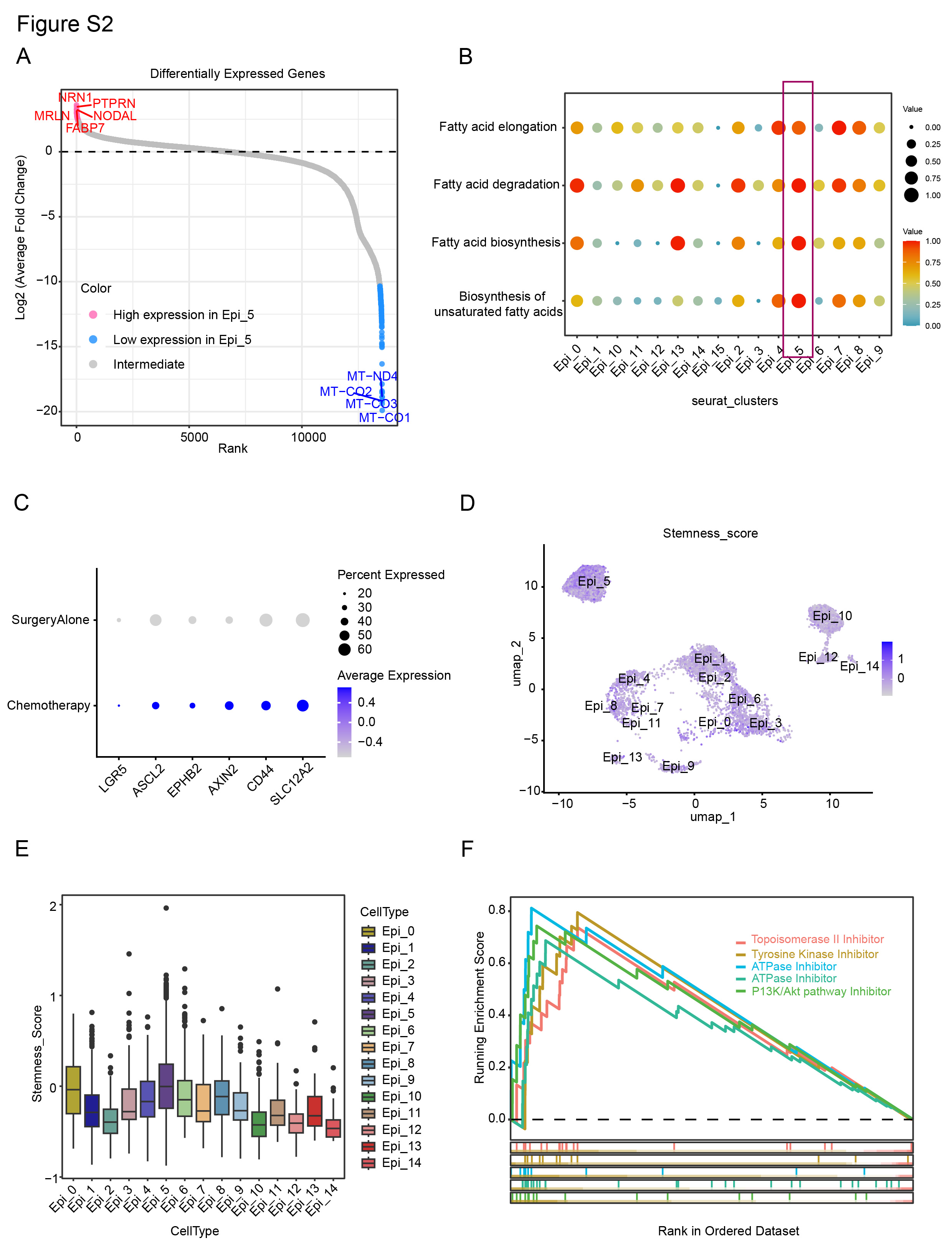

Supplement: Supplementary Figure 2 — Functional analyses of the Epi_5 cluster. (A) DEGs analyses between Epi_5 and all other tumor cell clusters, ranked by log2 fold-change. (B) Heatmap of fatty acid metabolism-related signaling pathways across all tumor cell clusters. (C) Dotplot showing CSCs in tumor samples before and after NAC. Color intensity indicates expression level while dot size represents the percentage of cells expressing each marker. (D, E) UMAP visualization (D) and Barplot showing the stemness scores of CSCs in various epithelial cell clusters. (F) GSEA plot displaying the top 5 drugs predicted to inhibit the Epi_5 cluster. [file Image2.jpg]

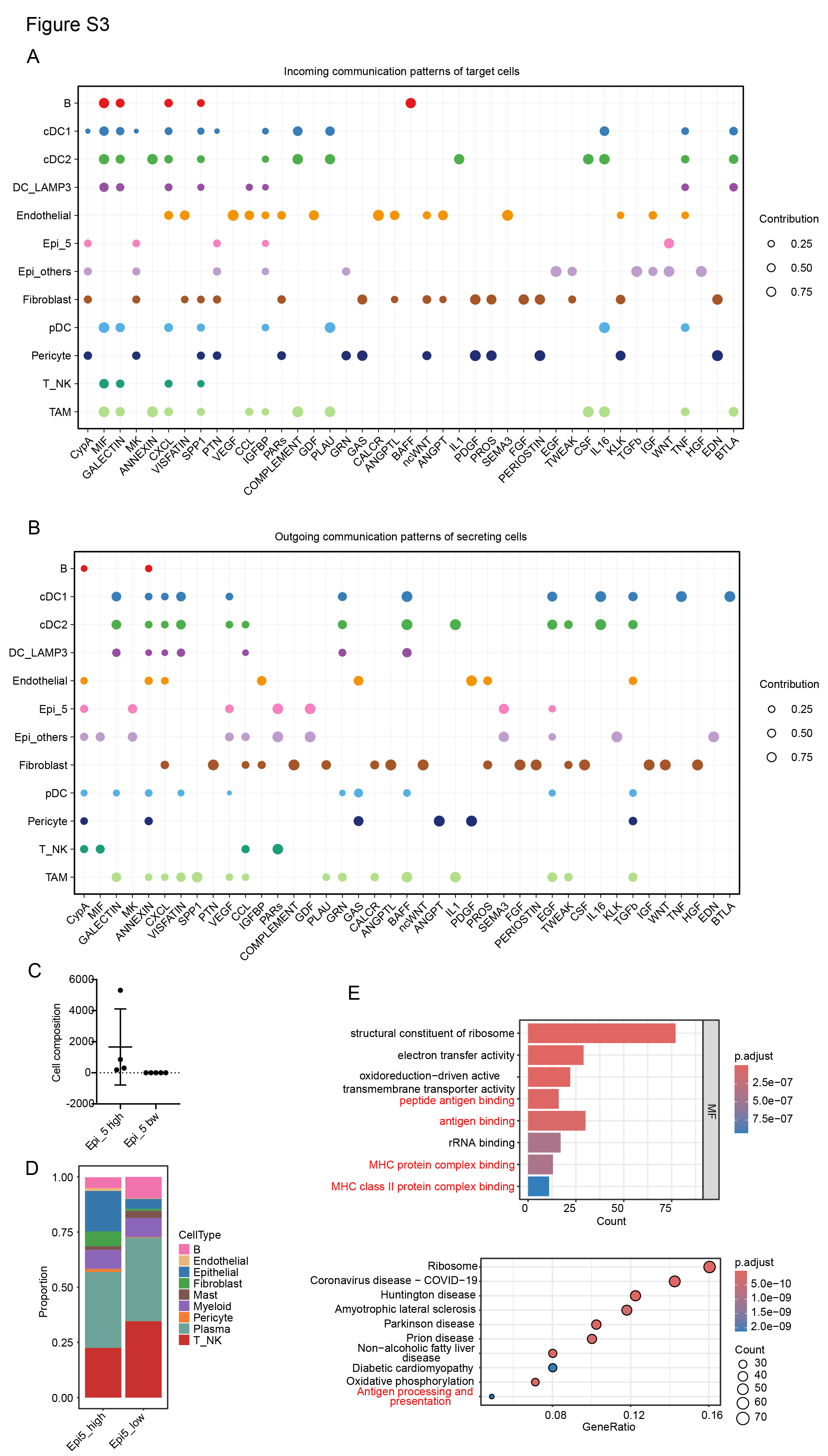

Supplement: Supplementary Figure 3 — Communication patterns of all cell clusters. (A) Incoming communication patterns of target cells, highlighting the number and types of signals received from other cell clusters. (B) Outgoing communication patterns of secreting cells, illustrating the number and types of signals sent to other cell clusters. (C) Dotplot illustrating the composition of Epi_5 cells categorized into high and low expression groups. (D) Proportions of major cell types within the Epi_5 high and low expression groups. (E) GO and KEGG enrichment analyses of differentially expressed genes in T_NK cells comparing Epi_5 high versus low expression groups. [file Image3.jpg]

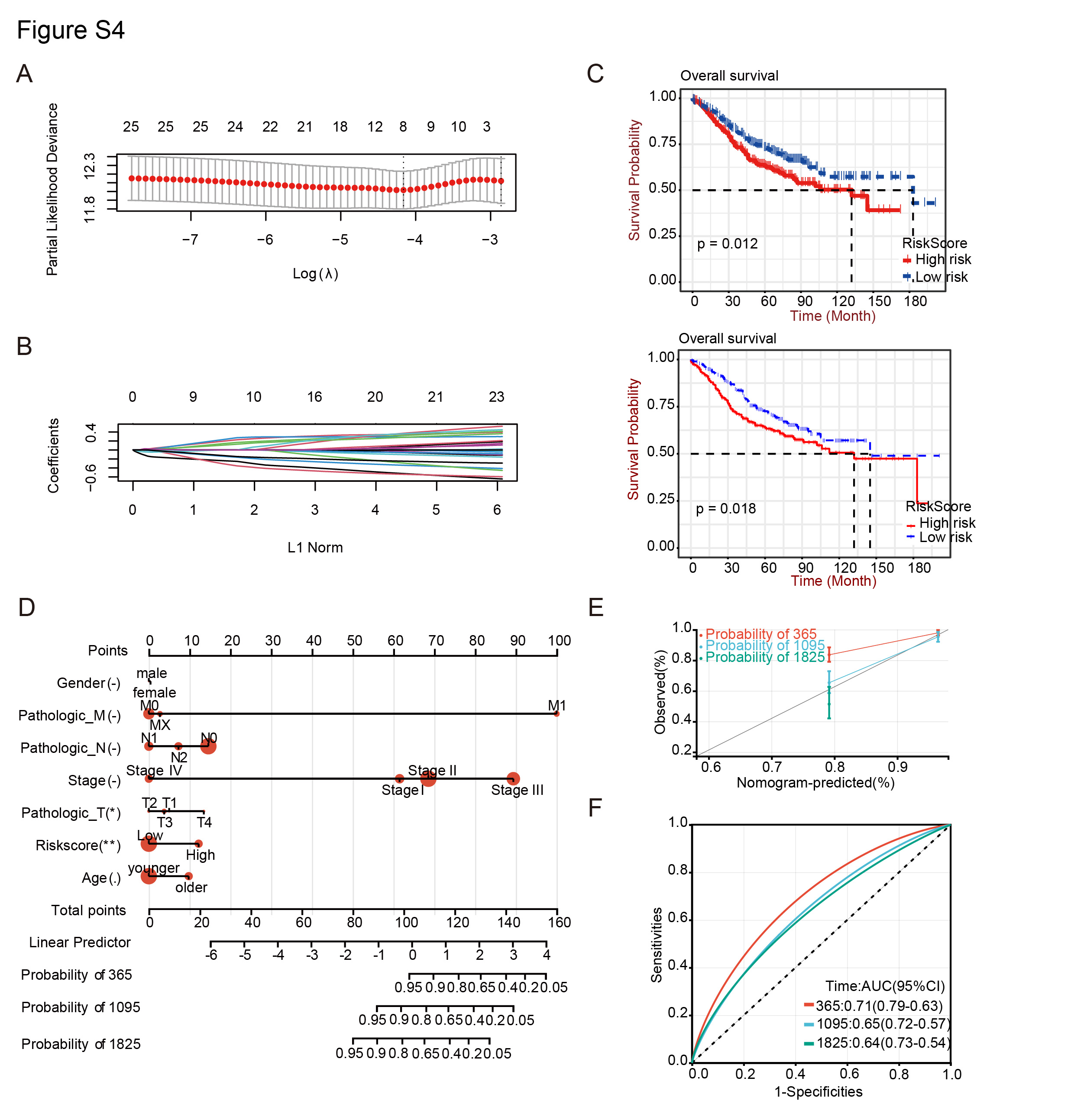

Supplement: Supplementary Figure 4 — Stability validation of the prognostic model. (A, B) LASSO regression analysis based on univariate Cox regression results to identify significant predictors. (C) Validation of the prognostic model using the GSE39582 and GSE17536 datasets. (D, E) Nomogram analysis of risk score and other clinical factors. (E) Nomogram showing the probability of survival at 365, 1095, and 1825 days. (F) ROC analysis of the prognostic model. [file Image4.jpg]

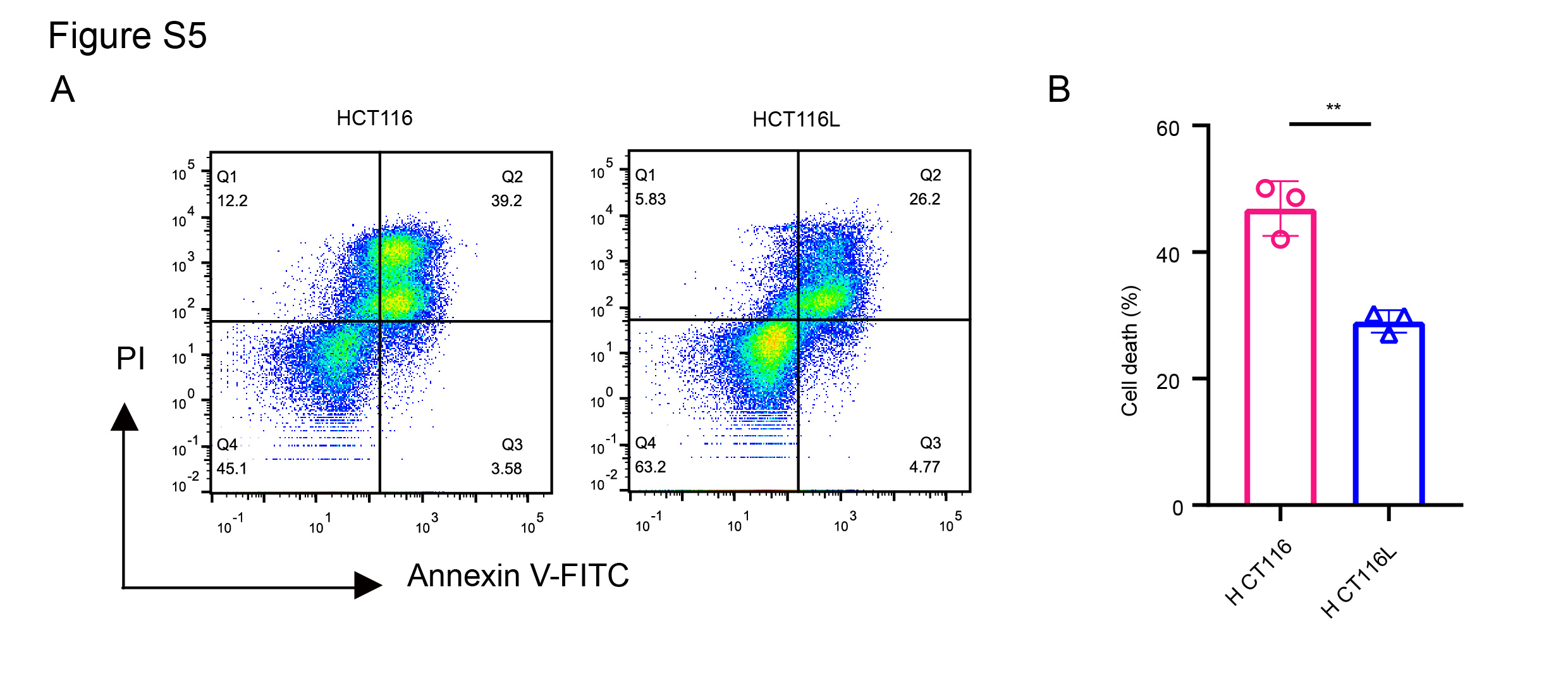

Supplement: Supplementary Figure 5 — Apoptosis analysis of HCT116 and HCT116L cell lines. HCT116 and HCT116L cell lines were treated with 40 μg/ml Oxaliplatin for 48 hours. Apoptotic cells were collected and analyzed using flow cytometry. Data were presented as mean ± SD. The statistical significance was determined by the Wilcoxon test (**p < 0.01). [file Image5.jpg]

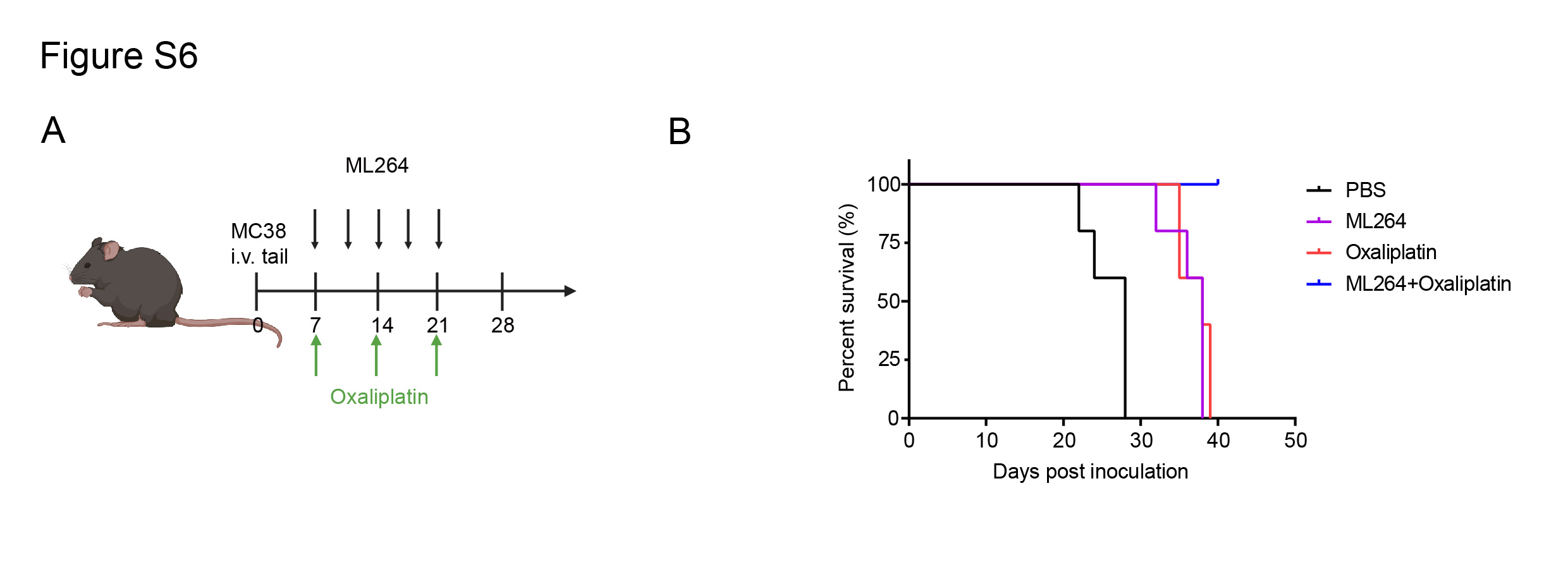

Supplement: Supplementary Figure 6 — The combination of ML264 and oxaliplatin suppresses lung metastasis and improves survival. (A) Schematic representation of the in vivo experimental design: C57BL/6N mice received 5×10^5^ MC38 cells via tail vein injection. Treatment groups received vehicle, oxaliplatin (5 mg/kg, once weekly), ML264 (25 mg/kg, twice weekly), or the combination. (B) KM survival curves demonstrating the impact of treatment regimens on mouse survival. The combination of ML264 and oxaliplatin significantly improved survival compared to individual treatments and vehicle control. [file Image6.jpg]
