# Supplementary material for: Targeting the KLF5/PI3K/AKT axis as a therapeutic strategy to overcome neoadjuvant chemoresistance in colorectal cancer
Source: Front Immunol. 2025 Jul 15;16:1593639. doi: 10.3389/fimmu.2025.1593639 (PMC12303937; doi:10.3389/fimmu.2025.1593639)
Supplement: Supplementary file 9 [file Table3.docx]

| **Characteristics** | **Alive(N=406)** | **Dead(N=102)** | **Total(N=508)** | **pvalue** | **FDR** |
| --- | --- | --- | --- | --- | --- |
| Age |  |  |  |  |  |
| Mean±SD | 65.75±12.57 | 69.01±12.56 | 66.40±12.62 |  |  |
| Median[min-max] | 67.50[31.00,90.00] | 71.50[34.00,90.00] | 68.00[31.00,90.00] |  |  |
| Gender |  |  |  | 0.74 | 0.74 |
| female | 193(37.99%) | 46(9.06%) | 239(47.05%) |  |  |
| male | 213(41.93%) | 56(11.02%) | 269(52.95%) |  |  |
| Pathologic_M |  |  |  | 5.2e-10 | 2.1e-9 |
| M0 | 327(64.37%) | 58(11.42%) | 385(75.79%) |  |  |
| M1 | 37(7.28%) | 35(6.89%) | 72(14.17%) |  |  |
| MX | 42(8.27%) | 9(1.77%) | 51(10.04%) |  |  |
| Pathologic_N |  |  |  | 1.3e-8 | 3.8e-8 |
| N0 | 256(50.39%) | 37(7.28%) | 293(57.68%) |  |  |
| N1 | 94(18.50%) | 26(5.12%) | 120(23.62%) |  |  |
| N2 | 56(11.02%) | 39(7.68%) | 95(18.70%) |  |  |
| Stage |  |  |  | 1.2e-11 | 5.9e-11 |
| Stage I | 87(17.13%) | 4(0.79%) | 91(17.91%) |  |  |
| Stage II | 165(32.48%) | 28(5.51%) | 193(37.99%) |  |  |
| Stage III | 116(22.83%) | 35(6.89%) | 151(29.72%) |  |  |
| Stage IV | 38(7.48%) | 35(6.89%) | 73(14.37%) |  |  |
| Pathologic_T |  |  |  | 1.0e-5 | 2.0e-5 |
| T1 | 13(2.56%) | 1(0.20%) | 14(2.76%) |  |  |
| T2 | 86(16.93%) | 4(0.79%) | 90(17.72%) |  |  |
| T3 | 271(53.35%) | 76(14.96%) | 347(68.31%) |  |  |
| T4 | 36(7.09%) | 21(4.13%) | 57(11.22%) |  |  |

**Table S3. Characteristics of CRC patients from the TCGA database.**
